# The application of SUDEP in forensic diagnosis: a mini review

**DOI:** 10.3389/fneur.2023.1169003

**Published:** 2023-04-26

**Authors:** Daming Sun, Qiang Wang

**Affiliations:** Forensic Science Center, East China University of Political Science and Law, Shanghai, China

**Keywords:** epilepsy, medical examiner, forensic diagnosis, molecular anatomy, SUDEP

## Abstract

In the epilepsy population, the risk of sudden death from epilepsy is rare but is ~24 times greater than the risk of sudden death from other causes. Sudden unexpected death in epilepsy (SUDEP) has been widely recognized in clinical studies. Despite its significance as a cause of death, SUDEP is rarely used in forensic practice. This review focuses on the forensic characteristics of SUDEP, analyzed the reasons for its underuse in forensic practice, and illustrated the prospect of establishing uniform diagnostic criteria for sudden unexpected death in epilepsy and molecular anatomy in aiding forensic diagnosis.

## Introduction

The methodology used in epidemiological studies and the type of epilepsy population assessed have a significant impact on the incidence of SUDEP. Disparities in SUDEP diagnostic criteria often affect the results. In the epilepsy population, the risk of sudden death from epilepsy is rare but is ~24 times greater than the risk of sudden death from other causes (1).

Sudden unexpected death in epilepsy is defined as a sudden, unexpected, witnessed or unwitnessed death, in a non-traumatic or non-drowning manner, with or without evidence of a seizure, excluding documented status epilepticus, in which there is no toxicological or anatomical cause of death (2). The risk of premature death is two to three times higher in a person with epilepsy than in the general population (3). There are three types of causes of death in people with epilepsy as follows: those unrelated to the disease; those brought on by the disease; and those caused directly by the disease (4, 5). The diagnosis of death related to epilepsy is mainly divided into two categories. The first type of death occurs in the state of seizures, and the other type is caused by seizures, such as traffic accidents caused by seizures in daily life, hospital treatment-related deaths, and even deaths resulting from seizures caused by fire (5, 6). There is growing evidence that SUDEP is one of the leading causes of death among people with epilepsy. This evidence demonstrates that it accounts for up to 17% of all deaths among this population (6).

## Current situation of SUDEP

### General characteristics of the deceased

According to the current study, young people are more likely to be affected by SUDEP (7, 8). Despite this being most likely, there has been no difference in the incidence of SUDEP in older adults. Evidence suggests that the incidence of SUDEP in older adults is underappreciated (9). A slight increase in the probability of dying from SUDEP was also observed in men compared to women (10).

### Circumstances of death

It is evident from previous studies that the majority of SUDEP deaths occurred at home (11–15), suggesting that all these individuals died while performing routine activities at home (7). A retrospective analysis of 67 cases revealed that the scenes of the deaths were similar, with 58 of them (87%) being discovered in their homes. A total of 38 cases (57%) were found dead on beds or couches. In terms of death posture, the body was discovered in a prone position majority of the time (16). Notably, most of these deaths were witnessed by no one (10, 12, 17, 18).

### Examination of corpses

There was evidence of recent seizure activity (tongue lacerations and contusions or superficial abrasions and contusions on the skin) in all cases (58%) that underwent autopsy. Visceral congestion and visceral edema were present in addition to pulmonary edema (11, 14, 19). Aside from structural brain lesions (17), aneurysms and tumors were the most common causes of epilepsy as determined by autopsy findings, followed by cerebrovascular malformations/aneurysms and brain tumors (10).

### Toxic (drug) substance test

According to a review of studies on postmortem physical and chemical detection, antiepileptic drugs (AEDs) and conventional poisons were determined as the cause of death in patients with SUDEP. The detection of antiepileptic drugs in postmortem cadaveric blood is classified into three major categories as follows: the presence of one antiepileptic drug, the presence of two antiepileptic drugs, and the presence of more than two antiepileptic drugs (16), with phenytoin being the most prevalent AED (10). Although there have been some evidence that the use of antiepileptic drugs may contribute to SUDEP, it is still unclear whether using more than one drug increases the risk of death. Several studies have suggested that the use of three AEDs may increase the risk of SUDEP by a factor of 10 when compared with monotherapy (20). Another study stated that SUDEP can occur even when only one anticonvulsant is administered at the therapeutic levels (18). Several possible explanations for the high mortality associated with combined therapy include disease severity, increased toxicity, highly variable, unpredictable, and complex drug interactions between antiepileptic drugs, the precipitation of central apnea following onset, and postural asphyxia caused by sedation-inducing combination therapy (6). The aforementioned studies have also shown that there is still a long way to go to clarify the pathological mechanism of SUDEP.

### Classification of cause of death

SUDEP is rarely used in forensic practice as a cause of death. According to a retrospective study of 104 epilepsy-related autopsy cases in Maryland, SUDEP was cited as the cause of death in only 7.7% of cases, while seizures or epilepsies were cited in 63.5% of cases (10). Additionally, another national survey of unexplained deaths caused by epilepsy revealed that, in most cases, SUDEP was not the medical examiner's preferred diagnosis on the death certificate, even when the cause of death could not be determined by autopsy and other possible causes of death had been excluded (21). It has been reported that SUDEP may be underreported on death certificates in Sweden and Wake County, North Carolina, with <30% of SUDEP cases listing SUDEP, seizures, or epilepsies as the cause of death (22).

## Discussion

### Forensic diagnostic criteria and identification points of SUDEP

Undoubtedly, SUDEP refers to a catastrophic death in people with epilepsy. Its incidence can have a substantial impact on an individual, their family, and society as a whole. Previous studies have primarily centered on risk factors. However, the underlying mechanisms and preventive measures are still unclear, and there is no evidence that these measures are effective (23). In addition, the incidence of SUDEP is also difficult to determine due to its dependence on the anatomy of the system for its diagnosis (24).

The following criteria must be satisfied to make a definitive diagnosis of SUDEP (23):

1. A person with epilepsy problems.
2. In the course of normal daily activities, the individual died (e.g., during sleep).
3. A sudden and unexpected death occurred within minutes of the accident.
4. There was no other medical condition that would predispose the individual to death.
5. Neither aspiration, suffocation, drowning, nor status epilepticus were known complications of the seizure episode that caused death.
6. Unknown cause of death.

An autopsy may not be able to identify the cause of death when all six criteria are met and a diagnosis of definite SUDEP is made (23). Probable SUDEP is considered when all six criteria are met but an autopsy is skipped. In addition, probable SUDEP is a very demanding diagnosis, which is because it generally serves as a substitute for an autopsy while also meeting the six diagnostic criteria listed above. If all six criteria are met even though an autopsy was not performed, SUDEP is considered probable. The possibility of SUDEP is taken into account when the death exhibits features of SUDEP, but an autopsy has not ruled out other causes of death. According to a retrospective analysis of nine SUDEP cases collected in China from January 2005 to June 2019, the following points should be considered when identifying SUDEP forensically. The following conditions were excluded as causes of death: epilepsy in young men; death while sleeping in prone or left lateral decubitus positions; substance abuse or mixed drug use before death; symptoms of asphyxia and urinary incontinence; unilateral hemorrhage in the neck muscle group and/or bilateral pectoralis minor muscle hemorrhage; pathological manifestations of respiratory depression and acute cardiac dysfunction; injuries, poisoning, and other organic diseases. Despite the small amount of data, SUDEP forensic identification may be guided by the summary of identification experience (16).

### The reasons for the low diagnostic rate of SUDEP

#### The uncertainty of SUDEP

It is not uncommon for medical examiners to be uncertain about the cause of death, as they must determine what they believe to be the most likely cause of death. Medical examiners usually perform an autopsy or forensic toxicology test when an autopsy cannot determine the cause of death. Medical examiners often neglect to determine whether epilepsy contributed to the death or was the cause of it (25).

SUDEP is rarely used as a cause of death in China because of the low autopsy rate of deaths. In most cases, patients with epilepsy are not autopsied due to a lack of consent from their families or the high cost of autopsies (21). A number of factors affect the rate of autopsy. The study showed that the family's acceptance of the autopsy and the cultural background of the individual are important factors. Families in some parts of China are reluctant to destroy the body of the deceased to show their respect for the deceased (26). Despite this, the study suggests that the autopsy rate in China will increase in the future as the use of autopsies becomes more popular and the need to clarify the cause of death increases. It is also possible to improve the certainty of medical examiners for SUDEP by collecting comprehensive medical history data of patients with epilepsy using big data technology and by collecting case-related information. As a result, SUDEP may be considered a more reliable cause of death to some extent.

#### Lack of awareness of SUDEP

SUDEP is not widely recognized among forensic pathologists, and/or forensic pathologists have differing philosophical opinions about how epilepsy-related deaths should be classified or labeled (27). Maryland medical examiners were surveyed to determine their acceptance of SUDEP. Based on the results of the survey, medical examiners preferred to use seizure disorder or epilepsy rather than a term that implies an uncertain cause when completing death certificates, despite the high rate of acceptance of SUDEP as a valid diagnosis (14/15 medical examiners) (10).

Pathology training appears to be useful in assisting medical examiners in diagnosing SUDEP when no cause of death was found at autopsy, according to a study on the awareness and use of SUDEP by coroners and medical examiners in urban and rural America (28). In recent years, forensic practice and scientific research have contributed to a more comprehensive understanding of SUDEP among neurologists (29) and medical examiners (28). However, compared to other causes of death, this improvement is still very low (30).

To improve the diagnosis of SUDEP as the cause of death, the study suggests that corresponding educational programs should be developed. A national SUDEP surveillance program and an international standard for the investigation and postmortem examination of SUDEP death scenes are also necessary to collect accurate statistics on SUDEP incidence (10).

### Application prospects of molecular autopsy

In 2016, the development and application of molecular anatomy were facilitated by high-throughput sequencing technology. Genetic factors contribute to the pathogenesis of epilepsy, which has a high prevalence in the population (31). Epilepsy is a condition caused by a dysfunctional ion channel. Additionally, a number of causal genes have been identified for epilepsy. As a result of the use of a mouse epilepsy model, KCNA1 knockout mice exhibited seizures, arrhythmia, increased vagal tone, and premature death (32), which was later validated in a human SUDEP case (33). According to another study, there is also evidence that CNVs on chromosome 15 are associated with autism, epilepsy, and SUDEP. In cases of SUDEP without autism, changes to CNVs in chromosome 15 can modify the risk of SUDEP (34). Finally, the hyperpolarization-activated cyclic nucleotide-gated ion channels HCN1-4 have been implicated in cardiac arrhythmias as well as epilepsy as they generate the cation (Na+ and K+)–triggered I_*h*_ depolarizing current that facilitates the generation of action potentials and spontaneous rhythmic activity in neurons and pacemaking cardiomyocytes (35–40).

As a result of expanding the SUDEP gene profile, medical examiners can recognize molecular anatomy more quickly and accurately. The detection of high-risk genes can help medical examiners make a definite diagnosis of SUDEP to a certain extent. However, there are also some problems in the practical application of molecular anatomies, such as non-standard molecular anatomy procedures, insufficient ability to interpret data, and the high cost of genetic testing (41). Sudden death can be effectively solved using molecular anatomy (42). Even though molecular anatomy has some shortcomings in practical application, we believe that molecular anatomy can be further developed and continues to play a role in the diagnosis of SUDEP with the development of science and technology and the improvement of the system, as well as the increasing frequency of international cooperation and interdisciplinary dialog in human and experimental translational research on sudden death.

## Conclusion

Despite reviewing the forensic examination of SUDEP, the authors were still unable to determine the typical characteristics of this disease. To establish the forensic diagnosis of SUDEP, a variety of indirect evidence must be presented. In practice, the presence of witnesses is a less common indication of SUDEP, but it is still a strong evidence of SUDEP.

The uncertainty of SUDEP is the reason for its low diagnostic rate. Nonetheless, there is a primary reason for solving this problem through systematic autopsy. The number of autopsies in China has to be increased as much as possible. Although molecular anatomy aids in the forensic diagnosis of SUDEP, there are a few limitations as well. Lack of awareness regarding this cause of death is another factor in the poor diagnosis rate of SUDEP. An international standard SUDEP postmortem examination protocol and systematic education are required to further improve medical examiners' knowledge of SUDEP.

In summary, this review first summarized the forensic characteristics of SUDEP and the reasons for the low diagnostic rate of SUDEP. Finally, it has been emphasized that the establishment of international standards and the application of molecular anatomy techniques will benefit the application of SUDEP in forensic diagnosis.

## Author contributions

QW performed the literature research, evaluated the literature, and wrote the manuscript. DS designed the study and participated in the writing and discussion of the results. All authors contributed to the manuscript and approved the submitted version.
